# A global study of the association of cesarean rate and the role of socioeconomic status in neonatal mortality rate in the current century

**DOI:** 10.1186/s12884-022-05133-9

**Published:** 2022-11-06

**Authors:** Abbas Alipour, Sedigheh Hantoushzadeh, Kamran Hessami, Maasoumeh Saleh, Mamak Shariat, Bahareh Yazdizadeh, Sepideh Babaniamansour, Azin Ghamari, Sepehr Aghajanian, Kamyar Moradi, Abolfazl Shirdel Abdolmaleki, Zahra Emami

**Affiliations:** 1 Department of Community Medicine, School of Medicine, Mazandaran University of Medical Sciences, Sari, Iran; 2 Department of Obstetrics and Gynecology, Maternal, Fetal and Neonatal Research Center, Tehran University of Medical Sciences, Valiasr Hospital, Tehran, Iran; 3 Maternal Fetal Care Center, Boston Children’s Hospital, Harvard Medical School, Boston, MA USA; 4 Department of Obstetrics and Gynecology, Tehran University of Medical Sciences, Shariati Hospital, Tehran, Iran; 5 Maternal, Fetal and Neonatal Research Center, Tehran University of Medical Sciences, Tehran, Iran; 6 Department of Epidemiology and Biostatistics, Knowledge Utilization Research Center, School of Public Health, Tehran University of Medical Sciences, Tehran, Iran; 7 School of Medicine, Islamic Azad University of Medical Science, Tehran, Iran; 8 Growth and development research center, Children’s Medical Center, Tehran University of Medical Sciences, Tehran, Iran; 9 Non-Communicable Diseases Research Center, Endocrinology and Metabolism Population Sciences Institute, Tehran University of Medical Sciences, Tehran, Iran; 10 Department of Community Medicine, School of Medicine, Alborz University of Medical Sciences, Karaj, Iran; 11 Student Research Center, Tehran University of Medical Sciences, Tehran, Iran; 12 School of Medicine, Shiraz University of Medical Sciences, Shiraz, Iran

**Keywords:** Caesarean section rate, Human development index, Neonatal mortality

## Abstract

**Introduction:**

Caesarean section (C/S) rates have significantly increased across the world over the past decades. In the present population-based study, we sought to evaluate the association between C/S and neonatal mortality rates.

**Material and methods:**

This retrospective ecological study included longitudinal data of 166 countries from 2000 to 2015. We evaluated the association between C/S rates and neonatal mortality rate (NMR), adjusting for total fertility rate, human development index (HDI), gross domestic product (GDP) percentage, and maternal age at first childbearing. The examinations were also performed considering different geographical regions as well as regions with different income levels.

**Results:**

The C/S rate and NMR in the 166 included countries were 19.97% ± 10.56% and 10 ± 10.27 per 1000 live birth, respectively. After adjustment for confounding variables, C/S rate and NMR were found correlated (*r* = -1.1, *p* < 0.001). Examination of the relationship between C/S rate and NMR in each WHO region resulted in an inverse correlation in Africa (*r* = -0.75, *p* = 0.005), Europe (*r* = -0.12, *p* < 0.001), South-East Asia (*r* = -0.41, *p* = 0.01), and Western Pacific (*r* = -0.13, *p* = 0.02), a direct correlation in America (*r* = 0.06, *p* = 0.04), and no correlation in Eastern Mediterranean (*r* = 0.01, *p* = 0.88). Meanwhile, C/S rate and NMR were inversely associated in regions with upper-middle (*r* = -0.15, *p* < 0.001) and lower-middle (*r* = -0.24, *p* < 0.001) income levels, directly associated in high-income regions (*r* = 0.02, *p* = 0.001), and not associated in low-income regions (*p* = 0.13). In countries with HDI below the centralized value of 1 (the real value of 0.9), the correlation between C/S rate and NMR was negative while it was found positive in countries with HDI higher than the mentioned cut-off.

**Conclusions:**

This study indicated that NMR associated with C/S is dependent on various socioeconomic factors such as total fertility rate, HDI, GDP percentage, and maternal age at first childbearing. Further attentions to the socioeconomic status are warranted to minimize the NMR by modifying the C/S rate to the optimum cut-off.

## Background

Cesarean delivery is a life-saving procedure with increasing prevalence for different emergency obstetrical conditions such as obstructed labor and considered as an essential healthcare service to reduce maternal and neonatal mortality [1–5]. World Health Organization (WHO) has reported the optimum C/S rate as 5–15% [6–9]. However, according to the literature, global C/S rate has risen from 12.4% to 18.6% during 1990 to 2014 [7]; C/S rate has surpassed the optimal threshold in several countries and in some cases accounts for 30% to 50% of total deliveries [1, 6, 10, 11]. While the high C/S rate in high-income countries has turned to a global concern, this procedure is hardly accessible in low-income countries even for cases with definite medical indication [12, 13].

C/S like any other surgery could give rise to a variety of complications and therefore, unnecessarily high C/S rate might cause increased neonatal mortality rate (NMR). NMR as a general health indicator is decreasing for the last two decades even though the decreasing trend has slowed down recently, especially in countries with middle- and high-income levels [6, 14–16]. Literature review shows that there is no direct association between C/S rate and NMR and seemingly, other factors might be involved.

A number of studies indicated that higher C/S rate is associated with higher rates of negative outcomes such as mortality for neonates [8, 11, 17–21]. This is while several other studies have suggested that C/S deliveries have made a great step in reducing NMR [22–24]. From an epidemiological perspective, disparities were evident in the relationship between NMR and C/S rate across different countries which raises the question whether other factors like socioeconomic status could affect this relationship.

Socioeconomic condition of a country has a great impact on the capability of the healthcare system, which is reflected in human development index (HDI) [1, 7, 19, 24–26]. It has been demonstrated that the financial motivation of C/S might be a possible reason for high C/S rates. For instance, C/S rate is higher in several lower middle-income countries due to significantly higher per case income from C/S compared to vaginal delivery as well as weak health payment system [5]. In support, NMR appears associated with the income levels of countries as those with higher gross domestic products (GDP) and HDI had lower NMR [26–28]. The high NMR due to C/S calls in some regions for greater attention to this issue. This study aimed to perform a multilevel logistic regression analysis to determine the impact of C/S rate on NMR considering different regions with different level of income.

## Material and methods

### Definitions

WHO categorized different countries by the geographical area into six regions as following: Africa (AFR), Americas (AMR), Eastern Mediterranean (EMR), Europe (EUR), South-East Asia (SEAR) and Western Pacific (WPR). World Bank region categorized the countries in to four income groups: one (high), two (upper-middle) and three (lower-middle) to four (low) [29]. C/S rate is defined as a percentage calculated by dividing the number of caesarean deliveries over the total number of live births in a year, multiplied by 100. MMR refers to the mortality rate of females during pregnancy or within 42 days of postpartum not related to the pregnancy period or the place of implantation per 100,000 live births in a year [30]. NMR refers to the mortality rate of neonate during first 28 days of life per 1000 live births in a year [31]. TFR refers to the total number of children born or likely to be born to a woman in her life if she survives all her childbearing [32]. HDI is defined as composite index reflecting three dimensions of human development including long healthy life, knowledge and access to resources for standard of living [33]. GDP refers to the total market value of final goods and services produced in each country each year [34]. Health expenditure per capita refers to the amount of money each country spend on both public and private sources on medical services and goods [35].

### Study design and data sources

This was a retrospective ecological study conducted at Maternal, Fetal & Neonatal Research Center, affiliated with Tehran University of Medical Sciences (TUMS), Tehran, Iran during March 2019 to March 2020. The protocol of this study was in accordance with declarations of Helsinki and approved by the ethics committee of TUMS (IR.TUMS.VCR.REC.1397.686). The list of study variables consisted of annual C/S rate, NMR, MMR, TFR, HDI, GDP percentage, health expenditure per capita and age of first childbearing.

The national survey data was collected from the multiple electronic databases published on the highest levels of health setting such as WHO and World Bank region. When needed, the data was extracted from health information system of each country. In order to achieve a comprehensive data, the list of WHO member states was used and searching process was completed to obtain maximum information on each variable using Google search engine. The available data of longitudinal type variables related to the C/S rate and NMR of different WHO regions was considered in 166 countries during 2000 to 2015. The countries with no data of C/S rate for at least one year in the study period were excluded. The data covered 85.12% of information of countries in the world. The official data reported from vital statistics or representative surveys by each country was used. The longitudinal data required for multilevel logistic regression consisted of the average annual private sector’s or public sector’s role from health expenditure, degree of risk, personal payment cost, level of income, usual payment method to the staffs, percentage of urban society, illiteracy percentage, percentage of access to safe drinking water, vaccination coverage rate, public fertility rate, prevalence of gestational diabetes mellitus, prevalence of Iron deficiency anemia and life expectancy of each country.

### Statistical analysis

All data were analyzed using SPSS version 24.0 (SPSS Inc., Chicago, IL., USA) and STATA version 14.0 (StataCorp. 2015. Stata Statistical Software: Release 14. College Station, TX: StataCorp LP.). Categorical variables were reported in percentage (%) while continuous data were represented as mean ± SD or median (Q25-Q75). The association between C/S rate and NMR was assessed using multilevel logistic regression model and geographical area and level of income were considered as two levels of analysis. The association between NMR and MMR, TFR, HDI, GDP percentage, health expenditure per capita and maternal age at first childbearing were assessed after adjusting for all other variables. *P* < 0.05 was considered statistically significant.

## Results

### The association between total C/S rate and NMR

In our study, the data of 166 countries that had available data of C/S rate of at least one year (from 2000 to 2015) were investigated. The C/S rate in these countries was 19.97% ± 10.56%. The NMR was 10 ± 10.27 per 1000 live birth. Other information related to these countries is shown in Table 1. Multilevel logistic regression showed that after adjusting for covariates, C/S rate is inversely associated with NMR and for each 1 percent elevation of C/S rate, NMR decreases by 1.1% (Table 2).

**Table 1:** Baseline information

**Variables**	**Median (Q25-Q75)**
C/S rate (*n* = 1079)	19.7 (13.51–26.9)
NMR (*n* = 1079)	5.1 (2.8–13.5)
MMR (*n* = 247)	17 (8–39)
TFR (*n* = 1058)	1.8 (1.46–2.4)
Maternal age at first childbearing (*n* = 1040)	28.7 (27.38–29.79)
HDI (*n* = 966)	0.78 (0.69–0.87)
GDP percentage (*n* = 1076)	6.94 (5.43–8.57)
Health expenditure per capita (*n* = 1076)	517.67 (140.48–2291.51)

*C/S* Cesarean Section, *NMR* Neonatal Mortality Rate, *MMR* Maternal Mortality Rate, *TFR* Total Fertility Rate, *HDI* Human Development Index, *GDP* Gross Domestic Products

**Table 2:** The relationship between NMR and each variable after adjusting for all other variables

**Variables**	**Regression coefficient** **(95% CI)**	**Standard error**	*p*-value^*^
C/S rate	-1.1 (-1.47, -0.73)	0.19	< 0.001
TFR	1.88 (1.28, 2.47)	0.3	< 0.001
HDI	-7.31 (-8.32, -6.3)	0.52	< 0.001
GDP percentage	0.5 (0.02, 0.98)	0.25	0.04
Maternal age at first childbearing	-0.24 (-0.46, -0.021)	0.11	0.03
The interaction between C/S rate and HDI	0.97 (0.37, 1.57)	0.31	0.002
The interaction between C/S rate and GDP	0.38 (-0.16, 0.92)	0.28	0.17

*CI* Confidence Interval, *C/S* Cesarean Section, *TFR* Total Fertility Rate, *HDI* Human Development Index, *GDP* Gross Domestic Products

^*^Refers to the relationship between NMR and each variable after adjusting for all other variables

### The association between C/S rate and NMR based on HDI

**Fig. 1:**
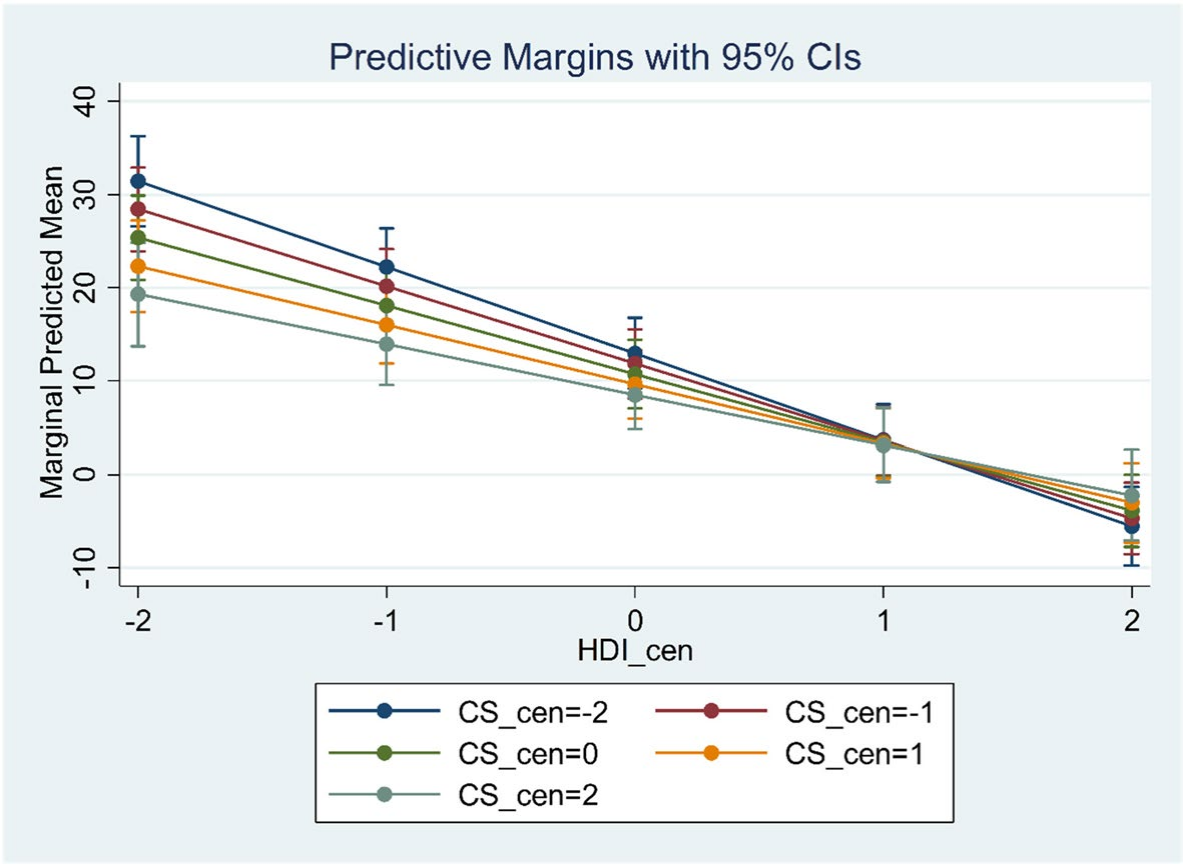
Correlation between C/S rate and NMR according to HDI

As evident in Fig. 1, in countries with HDI below the centralized value of 1 (the real value of 0.9), there is an inverse correlation between C/S rate and NMR while the correlation between C/S rate and NMR is positive in countries with HDI higher than the mentioned cut-off.

### The association between C/S rate and NMR in different WHO regions

The highest and lowest C/S rate were 27.83% ± 10.12% in AMR and 4.68% ± 3.71% in AFR. The highest and lowest NMR were 31.82 ± 8.36 and 6.82 ± 7.01 per 1000 live birth in AFR and EUR, respectively. Other information related to these countries is indicated in Table 3.

**Table 3:** The median of each outcome in the six different WHO regions

WHO region, Median (Q25-Q75)	C/S rate	NMR	MMR	TFR	Age of first childbearing	HDI	GDP percentage	Health expenditure per capita
AFR (*n*^a^ = 85)	3.6 (2.05–6)	31.4 (25.6–38.55)	450 (304–698.5)	5.22 (4.56–5.88)	29.14 (28.45–29.83)	0.46 (0.41–0.51)	5.21 (4.16–7.43)	34.89 (21.48–64.36)
AMR (*n* = 133)	27.1 (21.35–33.43)	9.4 (5.3–13)	61 (24–101)	2.11 (1.93–2.54)	27.71 (26.91–28.85)	0.72 (0.69–0.81)	6.77 (5.59–8.41)	377.67 (189.91–875.3)
EMR (*n* = 54)	19.35 (12.37–24.5)	9.5 (5.75–19.28)	24.5 (19.25–60.5)	2.96 (2.19–3.71)	29.84 (28.86–30.93)	0.74 (0.64–0.8)	4.09 (2.96–5.26)	314.83 (91.78–657.74)
EUR (*n* = 703)	19.33 (14.98–25.04)	3.6 (2.4–8.2)	10 (6–25)	1.59 (1.39–1.9)	28.65 (27.26–29.87)	0.82 (0.74–0.88)	7.31 (5.95–8.95)	923.27 (254.13–3017.1)
SEAR (*n* = 24)	12.35 (4.23–24.18)	21.6 (8.53–31.95)	203 (73.25–267.5)	2.42 (2.2–2.78)	28.17 (26.75–29.22)	0.6 (0.55–0.69)	3.38 (2.65–3.87)	48.62 (19.31–87.31)
WPR (*n* = 80)	29.45 (18.61–33.38)	3.3 (2.65–13.3)	18 (8–44)	1.92 (1.51–2.16)	29.34 (27.66–29.78)	0.87 (0.66–0.91)	6.82 (4.42–8.25)	1173.32 (89.75–2825.41)

*C/S* Cesarean Section, *NMR* Neonatal Mortality Rate, *MMR* Maternal Mortality Rate, *TFR* Total Fertility Rate, *HDI* Human Development Index, *GDP* Gross Domestic Products, *AFR* Africa, *AMR* Americas, *EMR* Eastern Mediterranean, *EUR* Europe, *SEAR* South-East Asia, *WPR* Western Pacific

^a^Number of countries in each continent

The association between C/S rate and NMR was assessed separately based on geographical area after adjustment of confounding variables in each region with the result available in Table 4. Four regions, including AFR, EUR, SEAR and WPR, there was an inverse significant association between C/S rate and NMR (*p* values < 0.05). AFR had the strongest association and for each 1 percent increase in C/S rate, NMR decreased by 0.75. On the other hand, the association between C/S rate and NMR was direct in AMR (*r* = 0.06, *p* = 0.04). Finally, no significant association was found between C/S rate and NMR in EMR (*p* = 0.88).

**Table 4:** The relationship between NMR and C/S rate in the six WHO regions

**Regions**	**Regression coefficient (95% CI)**	**Standard error**	*p*-value^*^
AFR	-0.75 (-1.28, -0.23)	0.26	0.005
AMR	0.06 (0.004, 0.13)	0.03	0.04
EMR	0.01 (-0.18, 0.21)	0.1	0.88
EUR	-0.12 (-0.16, -0.09)	0.02	< 0.001
SEAR	-0.41 (-0.72, -0.11)	0.15	0.01
WPR	-0.13 (-0.24, -0.02)	0.06	0.02

*CI* Confidence Interval, *AFR* Africa, *AMR* Americas, *EMR* Eastern Mediterranean, *EUR* Europe, *SEAR* South-East Asia, *WPR* Western Pacific

^*^Refers to the relationship between NMR and C/S rate after adjusting for all other variables

### The association between C/S rate and NMR among different income levels

In terms of wealth, the countries with highest income level had highest C/S rate with an average of 23.36% ± 7.04% while the low-income regions had the lowest C/S rate, 3.41% ± 2.27%. The highest and lowest NMR were 33.03 ± 7.78 and 3.12 ± 1.5 per 1000 live birth and were in low- and high-income regions, respectively. Other information related to these countries is reflected in Table 5. An inverse significant correlation was observed between C/S rate and NMR after adjusting the model for confounding variables in regions with upper-middle (*r* = -0.15, *p* < 0.001) and lower-middle (*r* = -0.24, *p* < 0.001) income level (Table 6). In contrast, there was a direct association between C/S rate and NMR (*r* = 0.02, *p* = 0.001) in the high-income region. Finally, no significant correlation was found between C/S rate and NMR in the low-income region (*p* = 0.13).

**Table 5:** The median of each variable according to four different levels of income groups

**Income level, Median (Q25-Q75)**	**C/S rate**	**NMR**	**MMR**	**TFR**	**Age of first childbearing**	**HDI**	**GDP percentage**	**Health expenditure per capita**
Group 1 (*n* = 540)	22.14 (17.4–28.77)	2.8 (2.2–3.6)	8 (5.5–13)	1.61 (1.39–1.9)	29.51 (28.46–30.23)	0.87 (0.83–0.9)	8 (6.8–9.29)	2280 (1040.11–4192.9)
Group 2 (*n* = 300)	21.39 (14.64–29.61)	9.65 (6.8–15.48)	35 (17.75–47.25)	1.88 (1.54–2.36)	27.3 (26.46–28.17)	0.73 (0.7–0.76)	5.89 (4.91–7.4)	277.67 (148.15–430.13)
Group 3 (*n* = 177)	8.85 (4.6–15.18)	18.1 (12.55–25.95)	71.5 (29.75–122.5)	2.6 (1.69–3.5)	28.12 (26.83–29.04)	0.63 (0.56–0.68)	5.66 (4.47–6.71)	67.4 (35.06–121.62)
Group 4 (*n* = 62)	2.95 (1.68–4.5)	31.65 (27.93–38.8)	450 (350.75–698.5)	5.31 (4.62–6.05)	29.21 (28.56–29.87)	0.44 (0.39–0.47)	5.45 (4.36–8.25)	25.12 (15.38–36.39)

*C/S* Cesarean Section, *NMR* Neonatal Mortality Rate, *MMR* Maternal Mortality Rate, *TFR* Total Fertility Rate, *HDI* Human Development Index, *GDP* Gross Domestic Products

**Table 6:** The association between NMR and C/S rate (extracted by regression analysis) according to income level

**Income level**	**Regression coefficient (95% CI)**	**Standard error**	*p*-value^*^
Group 1	0.02 (0.009, 0.04)	0.007	0.001
Group 2	-0.15 (-0.2, -0.1)	0.03	< 0.001
Group 3	-0.24 (-0.36, -0.13)	0.06	< 0.001
Group 4	-0.77 (-1.8, 0.24)	0.51	0.13

*CI* Confidence Interval, *AFR* Africa, *AMR* Americas, *EMR* Eastern Mediterranean, *EUR* Europe, *SEAR* South-East Asia, *WPR* Western Pacific

^*^Refers to the relationship between NMR and C/S rate after adjusting for all other variables

## Discussion

The current study determined the trend of NMR related CS in the world, adjusting for healthcare and economic related factors. Among all 166 countries, multilevel logistic regression showed that CS rate was inversely associated with NMR. However, at the region level, NMR was directly associated with CS rate in AMR, and inversely associated with CS rate in other regions. Moreover, NMR was directly associated with CS rate in high income region and was inversely associated with CS rate in middle income regions and there was no significant association in low-income regions.

The variation of CS rate not only could be explained based on the medical indications but also could reflect different health care systems and socioeconomic statuses. Identification the factors associated with CS rate and NMR is of paramount importance and increasing the evidence on this area may help making better decision for the healthcare system. In this regard, some studies showed that different HDI, GDP percentage, standardization of healthcare system, accessibility to CS, quality of obstetric cares, adequate medical care institutions and well-skilled attendants, TFR, sufficient surgical facilities and equipment, qualification of procedures and alteration of case payment affect the NMR and various studies confirmed that CS rate was directly associated with the levels of income [19, 36]. High income countries improve the health services coverage by enhancing HDI and GDP percentage which could contribute to decreased NMR especially in women with CS deliveries. Furthermore, enhancing the GDP percentage, HDI and health expenditure per capita could be better coverage for prenatal and postnatal cares [23, 28, 37, 38]. Annual NMR in developed countries changed more rapidly compared to less developed ones like sub-Saharan African countries; In 2010, Afghanistan, Congo, Mali, Pakistan and Somalia are the top five countries with the highest NMR but Iran, Bangladesh, Nepal, Botswana and Namibia had remarkable progress in reducing the NMR. NMR was inversely associated with the mount of government spend on health per capita, gross national income per capita and CS rate. TFR and total expenditure on health had direct association with NMR [39]. Therefore, income level of the country plays a key role in NMR and strategies regarding the reducing NMR should take this is issue into consideration.

Based on the Betrán et al. study, the global average CS rate was around 15% and it was highest in Latin American (29.2%) and lowest in African countries (3.5%). Beside the medical indications, CS rate may be associated with the economic factors of different areas. NMR was directly associated with CS rate in high income countries and was inversely associated with CS rate in low-income countries [38]. However, we found no association in low-income countries. In line with the previous study and our study, Villar et al. showed that CS rate in high income countries (Latin American countries) was directly associated with NMR [20]. Ye et al. assessed the association between NMR and CS rate in 19 high income countries in North and West Europe, North America, Australia, New Zealand, and Japan during 1980 and 2010, based on adjusted and unadjusted models for HDI and GDP. North America and Australia had the highest CS rates. HDI and GDP percentage were directly associated with CS rate and inversely associated with NMR. Increase CS from six to ten percent could significantly decrease the NMR in both models. Before adjusting for HDI and GDP, NMR reduction was only slightly while CS rate exceeded 20%. However, after adjusting for HDI and GDP, the flat curve was observed between NMR and CS rate while exceeded 10% [28]. Interestingly, Althabe et al. showed that CS rate was inversely associated with NMR in low income countries but the association was not statistically significant in middle and high income countries [23] and consistent with previous findings, Yisma et al. in Ethiopia (low income country) showed that between 2000 and 2016 the CS rate increased from 0.7% to 1.9% while the national NMR decreased from 4.8% to 2.9% [37]. Consistent with the results of the current study, Pradhan et al. showed that Nepal had 30% decline in NMR by three times increase in CS rate during 2000 and 2010. The decline of NMR related CS was associated with various socioeconomic factors such as the gross national income and significant decrease of TFR [26]. Contrary to the findings of these studies and also present study, Ochieng et al. conducted a study in Kenya and Tanzania (lower-middle income) and showed that NMR related CS was 4.4 times higher than NMR related VB in the poorest region (with lowest CS rate), after adjusting for antenatal visit and maternal and fetal risk factors. They stated that NMR related CS was 1.7 times higher than NMR related VBs in low socioeconomic regions of Kenya and Tanzania during 2014 to 2016 [19]. High NMR related CS could be explained by the fact that, the high proportion of CS rate in LMICs are the emergency type thus leads to increase the NMR in this area compared to the VBs. As Iran has the one of the highest CS rate in the Asia [13], Chaman et al. conducted a study among Iranian population in rural area and showed that NMR related CS was significantly higher than NMR related VB [40]. In contrast with their findings, a significant inverse association was observed in the middle-income region in our study.

Volpe et al. investigated the CS and NMR in an ecological study among 124 countries during 2000 to 2009. The global CS rate and NMR were 13.8% and 12 per 1000 live births. NMR was inversely associated with CS rate in the countries with CS rate of less than 15% CS rate. However, the association was not significant in the countries with more than 15% CS rate. The lowest NMR was observed in the countries with CS rate between 15 and 30% [41]. In contrast with this study, our study showed a significant association in high income region (high CS rate region) and non-significant association in low-income countries, which could be attributed to a longer period of evaluation and adjusting with the covariates. Pallasmaa et al. conducted a study among 12 delivery units in Finland (high income) during January to June 2005. They showed that the average of CS rate was 17.2%. Although, neonatal asphyxia and fetal distress rates were higher in any unit with higher CS rate but the association was not statistically significant [42]. MacDorman et al. found that the CS rate was increasing in the United States of America and compared to planned VB, NMR had 1.69 times higher in CS group with no complications, after adjusting for confounding variables [43]. Al Rifai. assessed the trend of CS rate in Egypt (lower-middle income) during 2005 and 2014 and showed that there was an inverse significant association between CS rate and NMR in any regions with less than 5% to 10% CS rate but CS rate of higher than 10% had no significant association with NMR [10]. However, in our study low-income region had the highest NMR and lowest CS rate but the difference was not statistically significant which may be due to the remarkable number of missing data in low-income region resulted from less developed healthcare information systems.

Although our study showed that high NMR in lower income regions was related to the inappropriate socioeconomic status and low CS rate, it is necessary to mention that in the low-income region, NMR related VB was also higher compared to high income region. Actually, lower income regions have poor quality birth system regardless of delivery methods. Betrán et al. showed that the global VB rate was 81.4%. Africa, Asia, Europe, Oceania and Latin American countries had the highest to lowest rate of VB. The VB had sharp decrease in Latin American countries and slow decrease in Africa [13]. VB’s adverse outcome is already higher in LMICs than high income countries. In this regard, A study among 13 high income countries showed that the frequency of postpartum hemorrhage (1.08% to 4.96%) and obstetric anal sphincter injury(1.12% to 3.73%), were higher among women with VB compared to those with CS [44]. However, kebede et al. showed that the incidence of postpartum hemorrhage in Ethiopia was 16.6% which was higher than Japan. They declared that the adverse effects disparities could be the result of the quality of healthcare services [45]. Abebe et al. reported similar NMR related VB (9.2%) or NMR related CS (12.6%) in Ethiopia. They declared that the complications could be related to the poor quality of obstetric care [46]. Decreasing the CS rate and subsequently increasing the rate of VBs in lower income regions could be a misconception for decreasing the NMR. Otherwise, improving the socioeconomic and healthcare related factors such as GDP percentage, HDI, health expenditure per capita, quality of insurance system for affording fees and access to adequate medications, health technology and equipment can be more decisive in low-income region [47–49].

One of the strengths of our study was that the association between NMR and CS rate was investigated in larger sample size and longer duration than previous studies. In addition, this may perhaps be the first study that assessed the association between NMR and CS rate in two different terms to achieve more comprehensive results. Our study also adjusted both healthcare related and socioeconomic factors in order to mitigate the effect of confounding variables and achieve real and better comparisons. Considering the results of the present study, the idea of reducing the NMR by reducing the CS rate was not “one size fits all” approach in any region and NMR related CS rate played as a function of countries’ socioeconomic status.

### Limitations

While this study assessed the association between the variables, the causal relation of the variables was not examined. Due to the lack of MMR information, we failed to MMR along with other variables, although this was one of primary goals. Accessing to the CS rate and GDP percentage of each country was roughly hard, especially in a long-term study. Therefore, utilization of electronic sources and data of similar studies in this topic by contacting the respective authors via electronic mail assisted the authors of this study to overcome the limitation. The NMR could be influenced by various possible factors like cultural and societal preferences favoring the use of C/S, socioeconomic status and quality of healthcare, expertise of the performing obstetrician, time of CS performance during labor, indication of CS, emergency of CS, type of hospital, that were not considered. The residual confounding factors can bias the association between CS rate and NMR.

## Conclusions

Although WHO recommends to decrease the CS rate in any region, our study showed that the disparities of HDI, GDP percentage, levels of income, health expenditure per capita and TFR in regions led to different results on the association between NMR and CS rate. This calls for greater attention to the issue and greater emphasizes on the importance of enhanced physicians’ knowledge in these areas.

## Data Availability

The data that support the findings of this study are available from Sedigheh Hantoushzadeh (hantoushzadeh@tums.ac.ir) but restrictions apply to the availability of these data, which were used under license for the current study, and so are not publicly available. Data are however available from the authors upon reasonable request and with permission of Sedigheh Hantoushzadeh (hantoushzadeh@tums.ac.ir).

## Abbreviations

C/S: Cesarean section
HDI: Human development index
GDP: Gross domestic product
NMR: Neonatal mortality rate
